# Screening for postpartum depression and risk of suicidality with obstetrical patients: a cross-sectional survey

**DOI:** 10.1186/s12884-023-05903-z

**Published:** 2023-09-04

**Authors:** Carlie Boisvert, Robert Talarico, Jasmine Gandhi, Mark Kaluzienski, Alysha LJ Dingwall-Harvey, Ruth Rennicks White, Kari Sampsel, Shi Wu Wen, Mark Walker, Katherine A. Muldoon, Darine El-Chaâr

**Affiliations:** 1 Faculty of Medicine, The Ottawa Hospital, University of Ottawa, Ottawa, Canada; 2 Clinical Epidemiology Program, Ottawa Hospital Research Institute, Ottawa, ON Canada; 3 Department of Obstetrics and Gynecology, University of Ottawa, Ottawa, Canada; 4 School of Epidemiology and Public Health, University of Ottawa, Ottawa, Canada; 5 International and Global Health Office, University of Ottawa, Ottawa, Canada; 6 Department of Obstetrics, Gynecology & Newborn Care, The Ottawa Hospital, Ottawa, Canada

**Keywords:** Postpartum depression, Suicide, COVID-19, Obstetrics, Pregnancy, Mental health, Self-harm, Suicidal ideation

## Abstract

**Background:**

Pregnancy is a vulnerable time where the physical and social stress of the COVID-19 pandemic affects psychological health, including postpartum depression (PPD). This study is designed to estimate the prevalence and correlates of PPD and risk of suicidality among individuals who gave birth during the COVID-19 pandemic.

**Methods:**

We surveyed individuals who gave birth at The Ottawa Hospital and were ≥ 20 days postpartum, between March 17 and June 16, 2020. A PPD screen consisted of a score ≥ 13 using the Edinburgh Postnatal Depression Scale. A score of 1, 2, or 3 on item 10 (“The thought of harming myself has occurred to me”) indicates risk of suicidality. If a participant scores greater than ≥ 13 or ≥ 1 on item 10 they were flagged for PPD, the Principal Investigator (DEC) was notified within 24 h of survey completion for a chart review and to assure follow-up. Modified Poisson multivariable regression models were used to identify factors associated with PPD and risk of suicidality using adjusted risk ratios (aRR) and 95% confidence intervals (CI).

**Results:**

Of the 216 respondents, 64 (30%) screened positive for PPD and 17 (8%) screened positive for risk of suicidality. The maternal median age of the total sample was 33 years (IQR: 30–36) and the infant median age at the time of the survey was 76 days (IQR: 66–90). Most participants reported some form of positive coping strategies during the pandemic (97%) (e.g. connecting with friends and family, exercising, getting professional help) and 139 (64%) reported negative coping patterns (e.g. over/under eating, sleep problems). In total, 47 (22%) had pre-pregnancy anxiety and/or depression. Negative coping (aRR:2.90, 95% CI: 1.56–5.37) and pre-existing anxiety/depression (aRR:2.03, 95% CI:1.32–3.11) were associated with PPD. Pre-existing anxiety/depression (aRR:3.16, 95% CI:1.28–7.81) was associated with risk of suicidality.

**Conclusions:**

Almost a third of participants in this study screened positive for PPD and 8% for risk of suicidality. Mental health screening and techniques to foster positive coping skills/strategies are important areas to optimize postpartum mental health.

**Supplementary Information:**

The online version contains supplementary material available at 10.1186/s12884-023-05903-z.

## Introduction

Shortly following the declaration of COVID-19 pandemic in March, 2020, there was global concern about how societal stress, uncertainty, isolation, and fear would negatively affect mental health [1–3]. Lessons from past epidemics including Severe Acute Respiratory Syndrome (SARS) and Middle East Respiratory Syndrome (MERS) found that the risk of infection and more severe physical symptoms are shigher among pregnant individuals [4, 5]. In addition to higher risk for more severe illness due to COVID-19 infection, the global pandemic adds further stressors to already existing pregnancy and post-partum mental health risks, including postpartum depression (PPD) and anxiety [6]. Pregnancy is a vulnerable time period where physical and social stress can negatively affect maternal physical and mental health, in turn affecting neonatal, infant, and longer-term childhood outcomes [7].

Mental health conditions, including PPD, have been reported to affect as many as 1 in 5 individuals during pregnancy and the first year post-partum [8]. The COVID-19 pandemic negatively impacted many social determinants that shape maternal mental health [9]. Household stressors such as income loss, social isolation from family and friends, limited access to community resources, and increased substance use were all identified as key risk factors amplified by the COVID-19 pandemic [10, 11]. Early surveys of the Canadian population found that over half of the respondents reported clinically relevant symptoms of anxiety in the first months of the COVID-19 pandemic [10]. Another Canadian population-level cross-sectional study found increases in physician visit rates for mental health among postpartum individuals during the first 9 months of the COVID-19 pandemic, suggesting the need for effective and accessible mental health care [12].

Suicide, one of the most severe outcomes among PPD cases, is a leading cause of maternal death during the 12 months following delivery, with thoughts of self-harm ranging from 1 to 14% [13–16]. An epidemiologic review of available evidence found that pregnant individuals are more likely to endorse suicidal ideation compared to the general population [17]. Additionally, during postpartum period, suicide attempts also involve higher means of lethality. A systematic review of the general population found that the rates of suicide attempts and deaths by suicide were higher during the COVID-19 pandemic compare to pre-COVID-19 estimates [18]. Risk factors for suicidality include PPD, anxiety, intimate partner violence, loneliness, lack of social support, and environmental exposure to crisis, disaster or conflict [19]. Individuals who are pregnant or postpartum during the COVID-19 pandemic face numerous risk factor increasing the risk of suicidality in addition to sustained and elevated prenatal anxiety [20].

With new COVID-19 variants, changing public health policies, and adaptations to healthcare provision, it is important to understand the impact of the COVID-19 pandemic on the mental health of those going through pregnancy/postpartum. The objectives of this study were to assess the prevalence of PPD and risk of suicidality among individuals who gave birth in the early phases of COVID-19 pandemic and identify the factors most strongly associated with PPD and risk of suicidality.

## Materials and methods

### Study setting and context

The study timeframe took place between March 17 and June 16, 2020 in Ottawa, Ontario, the capital of Canada with a population of 1.4 million [21]. At the time of the study there were 2,650 COVID-19 cases in Ottawa (2,240 recovered), 40,161 cases in Ontario (36,381 recovered), and 119,451 cases in Canada (112,709 recovered) [22]. The provincial government of Ontario declared a state of emergency on March 17, 2020 [23]. This included the shutdown of most public establishments (e.g. schools, childcare centers, libraries, recreational centers, restaurants, theatres, and concert venues) and most workplaces transitioned to remote work, where possible. There were no available vaccines at the time.

This study was conducted at The Ottawa Hospital (TOH), a multi-site tertiary-care facility with two obstetrical wards across the city. COVID-19 protocols were implemented by TOH Department of Obstetrics, Gynecology and Newborn Care (OBGYN), where all pregnant patients entering the hospital underwent symptomatic screening for COVID-19 at the hospital entrance and again upon entry to the Maternal and Newborn Care floor. The COVID-19 safety protocols included full personal protective equipment by all care providers. A partner or support person could only enter the hospital once (no in and out privileges) and only when the pregnant individual was in active labour [24].

### Study design and recruitment

This was a cross-sectional survey of TOH obstetrical patients. Participants were identified through the hospital birth records and contacted for a one-time survey if they had given birth since the 17th March 2020, were between 20 and 90 days postpartum, were 16 years of age or older, and consented to the institutions permission to contact program. Participants were contacted by phone by a trained research assistant (CB), all participants provided informed consent, and were then sent a link to the 10 min survey. All participants were provided with links to community resources for mental health, intimate partner violence, maternal support, or encouraged to contact their care providers for referrals.

This study and survey were designed in collaboration with our Patient Partner (O’Hare-Gordon). She was admitted to hospital for 5 weeks during the start of the COVID-19 pandemic, gave birth to pre-term twins who were admitted to the Neonatal Intensive Care Unit (NICU) for 19 days [25]. Her experience was invaluable to understanding the patient perspective and ensuring that the resources, study materials, and findings are relevant to the perinatal population.

### Outcome

The primary outcome of interest was a positive PPD screen, measured using the Edinburgh Postnatal Depression Scale (EPDS). The EPDS is the most reliable and widely used screening tool for PPD [26]. The 10-item scale ranges from 0 to 30 and a score of 13 or greater on the EPDS indicates a high likelihood of depression and further assessment/management is needed. A score of 1, 2, or 3 on item 10 (“The thought of harming myself has occurred to me”) indicates risk of suicidality. If a participant scores greater than ≥ 13 or ≥ 1 on item 10 they were flagged for PPD, the Principal Investigator (DEC, Maternal Fetal Medicine Specialist) was notified within 24 h of survey completion for a chart review and to assure follow-up. Detailed chart review was performed to make sure the patient had access to care for mental health with community provider or TOH perinatal mental health program to assure their safety. Messages to primary care providers were sent when applicable to ensure there was follow up for the positive screen for PPD and suicidality.

### Covariables

Demographic characteristics collected included age of mother (in years) and age of infant (in days) at the time of the interview. Maternal race/ethnicity was measured in five categories (White, South/South-East/East Asian, Middle Eastern, Black and other person of color), and a binary variable was derived to measure if the participant was a Person of Color (vs. White). Race and ethnicity categories were informed by Ottawa Public Health guidelines.

Participants were asked if they were born in Canada or immigrated. They indicated the language they were comfortable speaking and could select multiple answers (i.e., English, French, Other). Marital status compared those who were married/common law vs. single/other. Education was measured as having a college/university education or higher. Socio-economic status was measured through two variables; owned vs. rented dwelling, and gross household income below the Ottawa median (CAD $120,000) as determined by the Canadian Census [21].

COVID-19-related changes to childcare were measured as children stopped going to school or daycare, no changes to childcare, or no children. Variables measuring social isolation due to COVID-19 protocols included not having a baby shower, friends and family could not visit, or a family member who was planning to live with them postpartum could not come because of restrictions. Other COVID-19 pandemic variables included missing out on community resources because of pandemic protocols and having safety concerns about taking their baby outside the home.

Participants were asked to self-report about coping with the stress of the COVID-19 pandemic. Negative coping patterns including sleeping more or less than normal, over or under eating, or eating more unhealthy food, or violent/self-harming behaviour. Participants reported if they received counselling or treatment for pre-existing anxiety and depression. Participants reported on their stress and anxiety about receiving prenatal care, during labour and delivery and postpartum care. Breastfeeding variables included infant feeding method and were divided in planned method of feeding and actual feeding method in the past week (breast milk, formula, or both).

### Analysis

All analyses were conducted using SAS 9.4 [27]. In accordance with privacy guidelines, all cell sizes less than 5 were suppressed to ensure non-identification. Descriptive statistics include frequencies and percentages for categorical variables. Continuous variables were summarized using median and interquartile range (IQR). The characteristics were compared by calculating chi-square tests for categorical variables and Wilcoxon rank-sum test for continuous variables.

We used modified Poisson regression models to compute unadjusted risk ratios (RR) and adjusted risk ratios (aRR) 95% confidence intervals (CI) to identify potential risk factors associated with a positive PPD screen including: maternal age, infant age, parity, person of color, household income, negative coping strategies, pre-existing anxiety and/or depression, and feeding method.

For the analyses investigating risk of suicidality, only maternal age, infant age, and pre-existing anxiety and/or depression were chosen for the multivariable model to account for the smaller number of events. Risk factors were chosen to capture individual, interpersonal and household-level factors that influence risk of postpartum depression or suicidality.

There was at least one missing EPDS component for seven participants. We imputed missing components with the mean of the participant’s non-missing components to calculate the EPDS score. In the multivariable model, missing data for household income were imputed by multiple imputation using multivariable chained equations and models were averaged across 10 imputed datasets.

### Sensitivity analyses

To further investigate the role of suicidality, a sensitivity analysis was conducted. All participants with a positive screen for PPD (i.e. EPDS score of 13 or higher) and had no risk of suicidality (i.e. scored zero on item 10) were removed. This allowed for a comparison of those at risk of suicidality to those without any form of PPD (healthy control group). This study is a secondary analysis of complete survey data of 216 individuals. Based on an expected prevalence of 20% for PPD, with a confidence level of 95% the margin of error would be 5.33% using the available sample.

### Ethics

This study was approved by the Ottawa Health Sciences Network Research Ethics Board (Protocol number: 20200385-01 H).

## Results

During the study period, a total of 1568 obstetrical patients gave birth at TOH. Among those, 613 agreed to be contacted for research, 572 had valid phone numbers and were contacted, 302 consented to the study, 261started the survey with 216 having complete data for the analyses, giving an overall response rate of 42.6%.

There were 64 (29.6%) people with a positive screen for PPD and 17 (7.87%) with risk of suicidality. Table 1 compares the general characteristics of obstetrical patients with and without a positive screen for PPD. The maternal median age of the total sample was 33 years (IQR: 30–36) and the infant median age at the time of the survey was 76 days (IQR: 66–90). There were 69 (32%) who identified as a person of colour. There were 212 (98%) who spoke English and 112 (52%) had a combined household income above the Ottawa median. Most reported some form of positive coping strategies during the pandemic (97%) and 139 (64%) reported negative coping patterns. There were 136 (63%) who reported exclusive breast/chestfeeding. Detailed information on breast/chestfeeding practices are available in Appendix 1.

**Table 1:** General characteristics of obstetrical patients who gave birth during the first three months of the COVID-19 pandemic (n = 216)

Variables	PPD		Total	p-value
	Yes n = 64	No n = 152	n = 216	
Maternal Age in years (median, IQR)	33 (30–36)	33 (30–36)	33 (30–36)	0.756
Infant age in days at time of interview (med, IQR)	79 (68–89)	75 (65–90)	76 (66–90)	0.353
Month of Delivery in 2020				
March	10 (15.63%)	29 (19.08%)	39 (18.06%)	0.595
April	27 (42.19%)	55 (36.18%)	82 (37.96%)	
May	16 (25.00%)	48 (31.58%)	64 (29.63%)	
June	11 (17.19%)	20 (13.16%)	31 (14.35%)	
Nulliparous vs multiparous	34 (53.13%)	84 (55.26%)	118 (54.63%)	0.773
Person of color vs White	22 (34.38%)	47 (30.92%)	69 (31.94%)	0.619
Marital status: Married/common law vs single/other	61 (95.31%)	143 (94.08%)	204 (94.44%)	0.718
College Education or higher	54 (84.38%)	134 (88.16%)	188 (87.04%)	0.450
Born in Canada	45 (70.31%)	114 (75.00%)	159 (73.61%)	0.475
Speaks any of the following languages (all yes/no)				
Speaks English	62 (96.88%)	150 (98.68%)	212 (98.15%)	0.368
Speaks French	20 (31.25%)	54 (35.53%)	74 (34.26%)	0.545
Speaks another language	9 (14.06%)	20 (13.16%)	29 (13.43%)	0.859
Combined household income above Ottawa median (120,000+)				
Above Ottawa median	35 (54.69%)	77 (50.66%)	112 (51.85%)	0.337
Equal or below Ottawa median	25 (39.06)	55 (36.18)	80 (37.04)	
Missing^1^	-	-	24 (11.11)	
Dwelling is owned	38 (59.38)	113 (74.34)	151 (69.91)	0.029
Children’s care schedule during COVID-19				
Children stopped going to school or daycare	25 (39.06%)	59 (38.82%)	84 (38.89%)	0.886
Children’s schedule did not change	5 (7.81%)	9 (5.92%)	14 (6.48%)	
No children	34 (53.13%)	84 (55.26%)	118 (54.63%)	
Isolation due to COVID-19-19 restrictions (all yes/no)	63 (98.44%)	147 (96.71)	210 (97.22%)	0.481
No baby shower	35 (54.69%)	64 (42.11%)	99 (45.83%)	0.090
Family member couldn’t come to stay with me as planned	38 (59.38%)	70 (46.05%)	108 (50.00%)	0.074
Friends and family could not visit my new baby	55 (85.94%)	115 (75.66%)	170 (78.70%)	0.092
Missed out on community resources	58 (90.63%)	125 (82.24%)	183 (84.72%)	0.118
Safety concerns about taking my baby outside the home	59 (92.19%)	126 (82.89%)	185 (85.65%)	0.075
Positive coping strategies (yes/no)	64 (100.00%)	145 (95.39%)	209 (96.76%)	0.081
Connecting with friends/family	34 (53.13)	95 (62.50)	200 (92.59)	0.650
Exercising	35 (54.69)	91 (59.87)	126 (58.33)	0.469
Getting professional help	12 (18.75)	13 (8.55)	25 (11.57)	0.096
Negative coping patterns (yes/no)	54 (84.38%)	85 (55.92%)	139 (64.35%)	<0.001
Sleeping more or less than normal	40 (62.50%)	62 (40.79%)	102 (47.22%)	0.013
Over or under eating, or eating more unhealthy foods	39 (60.94%)	55 (36.18%)	94 (43.52%)	0.004
Acting violently, including self-harm^1^	-	-	5 (2.31)	0.607
Had any stress/anxiety about receiving	55 (85.94)	102 (67.11)	157 (72.69)	0.005
Prenatal care (yes/no)	51 (79.69%)	85 (55.92%)	136 (62.96%)	<0.001
Labour and delivery (yes/no)	33 (51.56%)	52 (34.21%)	85 (39.35%)	0.017
Post-partum care (yes/no)	38 (59.38%)	67 (44.08%)	105 (48.61%)	0.040
Feeding method in the past week				
Breastmilk only	33 (51.56%)	103 (67.76%)	136 (62.96%)	0.148
Formula	14 (21.88%)	22 (14.47%)	36 (16.67%)	
Combined	15 (23.44%)	25 (16.45%)	40 (18.52%)	

1. Small cells ≤ 5 are suppressed

NB. The study took place between March 16th to June 16th, 2020

Table 2 shows the EPDS score and mental health characteristics of the study participants, including pre-pregnancy mental health conditions. The median EPDS score was 8 (IQR 4–13) for the entire sample, 15 (IQR 13–17) for those with a positive PPD screen, and 17 (IQR14-19) for those with a positive screen for risk of suicidality. In total, 47 (22%) had pre-pregnancy anxiety and/or depression.

**Table 2:** Mental health characteristics of obstetrical patients who gave birth during the first three months of the COVID-19 pandemic (n = 216)

Variables	PPD		Suicidality		Total
	Yes (n = 64)	No (n = 152)	Yes (n = 17)	No (n = 199)	n = 216
EPDS Score (med, IQR) ^1^	15 (13–17)	6 (3–9)	17 (14–19)	8 (4–12)	8 (4–13)
Risk of suicidality ^2^	17 (26.56%)	0 (0.00%)	17 (100%)	0 (0.00%)	17 (7.87%)
Anxiety sub-scale^3^	47 (73.44%)	15 (9.87%)	13 (76.47%)	49 (24.62%)	62 (28.70%)
Mental health					
Pre-pregnancy anxiety or depression (yes/no)	23 (35.94%)	24 (15.79%)	8 (47.06%)	39 (19.60%)	47 (21.76%)
Pre-pregnancy anxiety	22 (34.38%)	21 (13.82%)	8 (47.06%)	35 (17.59%)	43 (19.91%)
Pre-pregnancy depression	16 (25.00%)	13 (8.55%)	7 (41.18%)	22 (11.06%)	29 (13.43%)

NB. The study took place between March 16th to June 16th, 2020

1. EPDS – Edinburgh Postnatal Depression Scale

2. A score of 1, 2, or 3 on item 10 on the EPDS about suicidal thoughts was flagged as risk of suicidality

3. The Anxiety sub-scale is calculated by questions 3,4,5 of the EPDS

Table 3 displays the unadjusted and adjusted modified Poisson regression model to assess factors associated with a positive PPD screen. Negative coping patterns (aRR: 2.90, 95% CI: 1.56 to 5.37) and pre-existing anxiety and/or depression (aRR: 2.03, 95% CI: 1.32 to 3.11) were significantly associated with a positive PPD screen. There was no significant associations with maternal age at delivery, age of infant at survey completion, nulliparity, ethnicity, household income, or exclusive breast/chestmilk feeding in the past week.

**Table 3:** **Unadjusted and adjusted modified Poisson regression models to assess factors associated with post-partum depression (n = 216)**

Co-Variables	RR (95% CI)	p-value	aRR (95% CI)	p-value
Maternal age at delivery (yrs)	1.00 (0.95–1.05)	0.929	1.02 (0.96–1.08)	0.513
Age of infant at survey completion (days)	1.00 (0.99–1.02)	0.422	1.00 (0.99–1.01)	0.945
Parity	0.94 (0.62–1.42)	0.773	0.94 (0.62–1.44)	0.778
Person of color	0.90 (0.58–1.38)	0.617	0.74 (0.47–1.16)	0.190
Equal or below household income under median for Ottawa	1.00 (0.65–1.53)	1.000	0.77 (0.49–1.22)	0.276
Negative coping	2.99 (1.62–5.53)	< 0.001	2.77 (1.49–5.13)	0.001
Pre-existing anxiety and/or depression	2.01 (1.36–3.00)	< 0.001	1.85 (1.18–2.91)	0.007
Feeding Method in the past week				
Breast milk only	0.62 (0.38–1.03)	0.068	0.77 (0.47–1.25)	0.287
Combined	0.96 (0.54–1.71)	0.901	0.88 (0.52–1.48)	0.626
Formula	(ref)		(ref)	

NB. The study took place between March 16th to June 16th, 2020

Appendix 2 has the general characteristics of obstetrical patients with and without risk of suicidality. Descriptively, the two groups were similar by many demographic factors (e.g. age, education status, language etc.) Table 4 displays the unadjusted and adjusted modified Poisson regression models for factors associated with risk of suicidality. Only three variables were entered in the model because of fewer events of risk of suicidality. Pre-existing anxiety and/or depression was significantly associated with risk of suicidality (aRR: 3.16, 95% CI:1.28–7.81). There was no independent association risk of suicidality with maternal or infant age

**Table 4:** Unadjusted and adjusted modified Poisson regression models to assess factors associated with risk of suicidality (n = 216)

Co-Variables	RR (95% CI)	p-value	aRR (95% CI)	p-value
Maternal age at delivery (yrs)	1.04 (0.96–1.15)	0.307	1.06 (0.96–1.16)	0.270
Age of infant at survey completion (days)	1.01 (0.99–1.03)	0.222	1.01 (0.99–1.03)	0.367
Parity	0.45 (0.17–1.18)	0.105		
Person of color	0.53 (0.21–1.31)	0.168		
Household income under	1.40 (0.55–3.57)	0.482		
Negative Coping Strategies	4.15 (0.98–17.69)	0.054		
Pre-existing anxiety and/or depression	3.20 (1.30–7.83)	0.011	3.16 (1.28–7.81)	0.013
Feeding Method in the past week				
Breast milk only	0.71 (0.20–2.53)	0.592		
Combined	1.50 (0.39–5.84)	0.559		
Formula	(ref)			

NB. The study took place between March 16th to June 16th, 2020

In the sensitivity analysis, the 47 cases of a positive PPD screen alone were removed to compare those with risk of suicidality (n = 17) those without any form of PPD (n = 169) (healthy controls). The sensitivity analysis showed no changes in the estimates. The results of the sensitivity analysis are available in Appendix 3 and 4.

## Discussion

Our study found that almost 30% of obstetrical patients who gave birth during the first three months of the COVID-19 pandemic screened positive for PPD and almost 8% screened positive for risk of suicidality. Pre-existing mental health issues and negative coping patterns (e.g. sleeping and eating problems, and rare instances of self-harm/violence) were the factors most strongly associated with screening positive for PPD. This highlights the importance of comprehensive inquiry into the background of pre-existing mental health issues and the need for additional resources to help people actively cope with general as well as pandemic-related stress. Differences by maternal age, infant age, parity, race/ethnicity, income, or breast/chestfeeding practices were not significantly associated with screening positive for PPD or risk of suicidality in this sample.

The results from this study are comparable to the results of a systematic review of 54 studies on PPD during the COVID-19 pandemic, identifying an overall prevalence of 33% [28]. These estimates are almost three times higher than the prevalence of PPD before the COVID-19 pandemic, estimated at 12% [29]. There is considerable variability in how PPD is measured, ranging from self-reported symptoms, screening, to clinical diagnosis. While useful for research purposes, The Canadian Task Force on Preventive Health Care recommends against individual screening instruments for PPD (e.g. EPDS), and instead states that routine clinical monitoring of mental health and well-being offers more clinical benefit and is time effective [30].

Of importance to our research team and the study participants, anyone who screened positive on these scales had a detailed chart review and were confirmed to be connected to primary care provider or perinatal mental health by our teams Maternal Fetal Medicine specialist (DEC) for clinical follow-up, when applicable a message was sent to their care provider as a flag. A strength of our study is the use of the validated EPDS screening tool [26]. While not diagnostic, it is a strong indicator of PPD, signaling the need for clinical follow-up for each patient. In our study, over 35% of individuals who screened positive for PPD had pre-existing depression or anxiety that they had received counselling or medical treatment for in the past. Identifying pre-existing mental and physical health conditions during the prenatal check-ups is an important protective step to either prevent the development of PPD or help prepare people at higher risk of PPD with healthy coping strategies and resources [31, 32].

A meta-analysis of studies in the general population found that the prevalence of depression during COVID-19 pandemic was 28% [33] compared to 17% from a meta-analysis of studies conducted before the pandemic [34]. The restrictions associated with the COVID-19 protocols interrupted many services (e.g. office closures, reduced hours, shifts to virtual care etc.) all affecting access to assessment, treatment and support for PPD.

Of concern, almost 8% of participants in this study expressed thoughts of suicidality. The strongest risk factor for risk of suicidality was also pre-existing anxiety and/or depression, with 47% of the participants at risk of suicidality reporting pre-existing mental health conditions compared to 36% of those with a positive PPD screen, and 20% for the participants without PPD or risk suicidality. It is well established that pre-existing mental health conditions increase the risk of suicidality during pregnancy and postpartum [13, 17, 35]. Pre-COVID-19 studies have estimated that 20% of postpartum maternal deaths are attributed to suicide [35]. In the general population, suicide attempts and death by suicide have risen during the COVID-19 pandemic, [18], however, it is yet to be determined how this will change for the perinatal population. Our results suggest that maternal mortality due to suicide could increase during the COVID-19 pandemic and careful screening and monitoring is needed to reduce the negative impact that COVID-19 related stress can have on maternal and postpartum mortality. Moreover, effective interventions for PPD, such as psychosocial interventions (e.g. peer support, non-directive counselling by clinical staff or peers), psychological interventions provided by qualified health professionals (e.g. cognitive behavioural therapy, interpersonal therapy, group therapy, couples therapy), or medication management, are important to provide when an individual with PPD or risk of suicidality is identified [36].These interventions can also mitigate negative effects of PPD on the infant, other children, or the non-birthing partner. Given the high levels or mortality due to postpartum suicide in general, the timeliness of accessing effecting services is also paramount.

It is encouraging to see that most participants in this sample reported some form of positive coping skills (e.g. connecting with friends and family, exercising, getting professional help etc.). However, almost 85% of those who screened positive for PPD and 88% of those with risk of suicidality reported negative coping patterns (e.g. sleeping and eating problems, and in rare cases self-harm or acting violently), compared to 56% of those without a positive screen for PPD or risk of suicidality. Negative coping was significantly associated with a positive screen for PPD after adjusting for co-variables. Due to small cell sizes, we could not run adjusted models on coping and risk of suicidality, however it was significantly associated at the unadjusted level. Sleep quality and eating patterns are known to change with gestational age and during the postpartum period, increasing the risk of PPD [37]. Additionally, they are also symptoms of PPD. For that reason, we specified coping patterns related to the stress of the COVID-19 pandemic. While positive coping strategies will not ‘cure’ negative coping patterns, building healthy coping skills, practicing mindfulness, cognitive restructuring, physical movement and exercise, being in nature, and social connections will reduce susceptibility to biological/social stressors over which there is little control (e.g. COVID-19 pandemic, pre-existing mental health conditions etc.).

Social support can buffer the effects of prenatal stress and has been shown to mitigate the impacts of PPD symptoms [38]. The COVID-19 public health restrictions on gatherings, and requirements to physically distance and stay at home can increase isolation and the chance of negative coping going unnoticed. Community resources, such as new parent groups or postnatal fitness classes, are useful for connecting people in the postpartum period to share experiences and building meaningful and supportive friendships. It is also important not to minimize the effect of the loss of social support one can build when starting a family. Social conditions, social relationships, and supportive neighbourhood environments are important determinants of health during pregnancy and the postpartum period [39], Further work to optimize the availability and effectiveness of community resources through virtual means could be helpful for future epidemics as well as more broadly to increase accessibility.

### Limitations

Self-selection bias is present in our study as we do not have information on those who declined to participate. Participants were contacted between 20 and 90 days postpartum, reducing recall bias. Demographically, our sample is older than the typical perinatal population, had higher socio-economic status and higher education. It is unclear if participants with higher risk of PPD would be more or less motivated to participate and complete the survey. The EPDS is a validated screening tool and not a diagnostic tool, therefore the study cannot give information on confirmed depression and suicidality diagnoses. Suicidality was assessed specifically by item 10 from the EPDS about self-harming impulses, however, there are other dimensions of suicidality that the survey did not assess for a more precise risk assessment, such as prior psychiatric illness and suicide attempts [35]. As we do not have a pre-COVID-19 comparison group, we must rely on studies of similar population for comparison purposes. As our institution is a tertiary care centre, there are potentially higher risk pregnancies that may limit generalizability to the general obstetrical population.

## Conclusion

To conclude, this study has documented that 30% of those who gave birth in the early months of the COVID-19 pandemic screened positive for PPD and 8% screened positive for risk of suicidality. Pre-existing mental health conditions and negative coping patterns were strongly associated with PPD and risk of suicidality. These findings emphasize the importance of routine mental health and wellness check-ins at prenatal and postpartum visits. Both clinical health services and support to develop positive skills and strategies for coping with stress and challenges during the perinatal period are important protective factors.

### Electronic supplementary material

Below is the link to the electronic supplementary material.

Supplementary Material 1

## Data Availability

The datasets generated and analyzed during the current study are not publicly available due to The Ottawa Hospital privacy protocols, but with a data sharing agreement, de-identified data, the data dictionary, and ethics protocol are available from the corresponding author.
