# Supplementary material for: Screening for postpartum depression and risk of suicidality with obstetrical patients: a cross-sectional survey
Source: BMC Pregnancy Childbirth. 2023 Sep 4;23:635. doi: 10.1186/s12884-023-05903-z (PMC10478309; doi:10.1186/s12884-023-05903-z)
Supplement: Supplementary file 1 — Supplementary Material 1 [file 12884_2023_5903_MOESM1_ESM.docx]

**Appendix Table 1. Breastfeeding practices among participants comparing PPD among those who gave birth during the beginning of the COVID-19 pandemic (n=216)**

| **Variables** | **PPD** | | **Total** | **p-value** |
| --- | --- | --- | --- | --- |
|  | Yes  N=64 | No  N=152 | N= 216 |  |
| **Planned method of feeding** |  |  |  |  |
| Breast milk only | 48 (75.00%) | 118 (77.63%) | 166 (76.85%) | 0.249 |
| Formula only | 4 (6.25%) | 2 (1.32%) | 6 (2.78%) |  |
| Combined | 11 (17.19%) | 29 (19.08%) | 40 (18.52%) |  |
| **Actual method of breast feeding in the last week** |  |  |  |  |
| Breast milk only | 33 (51.56%) | 103 (67.76%) | 136 (62.96%) | 0.148 |
| Formula only | 14 (21.88%) | 22 (14.47%) | 36 (16.67%) |  |
| Combined | 15 (23.44%) | 25 (16.45%) | 40 (18.52%) |  |
| **Any difficulties breastfeeding (yes/no)** | 46 (71.88%) | 84 (55.26%) | 130 (60.19%) | 0.023 |
| Nipple and/or breast pain | 31 (67.39%) | 54 (64.29%) | 85 (65.38%) | 0.722 |
| Inadequate milk volume | 26 (56.52%) | 40 (47.62%) | 66 (50.77%) | 0.332 |
| Mastitis | 7 (15.22%) | 12 (14.29%) | 19 (14.62%) | 0.886 |
| Infant poor weight gain | 13 (28.26%) | 17 (20.24%) | 30 (23.08%) | 0.299 |
| **Did you get help for breastfeeding problems (yes/no)** | 35 (54.69%) | 60 (39.47%) | 95 (43.98%) | 0.040 |
| **If yes, from who:** |  |  |  |  |
| Family doctor/nurse practitioner/midwife/doula | 17 (48.57%) | 26 (43.33%) | 43 (45.26%) | 0.621 |
| Lactation consultant | 22 (62.86%) | 36 (60.00%) | 58 (61.05%) | 0.783 |
| Public health | 3 (8.57%) | 7 (11.67%) | 10 (10.53%) | 0.635 |
| Specialized clinic | 21 (60.00%) | 36 (60.00%) | 57 (60.00%) | 1.00 |
|  |  |  |  |  |

NB. The study took place between March 16th to June 16^th^, 2020.

**Appendix Table 2: General characteristics of obstetrical patients comparing risk of suicidality among those who gave birth during the first three months of the COVID-19 pandemic (n=216)**

| **Variables** | **Risk of suicidality** | | **Total** | **p-value** |
| --- | --- | --- | --- | --- |
|  | Yes  n=17 | No  N=199 | n=216 |  |
| Maternal Age in years (median, IQR) | 33 (31-36) | 33 (30-36) | 33 (30-36) | 0.437 |
| Infant age in days at time of interview (med, IQR) | 80 (71-91) | 76 (65-89) | 76 (66-90) | 0.264 |
| Month of Delivery in 2020 |  |  |  |  |
| March | 5 (29.41%) | 34 (17.09%) | 39 (18.06%) | 0.302 |
| April | 5 (29.41%) | 77 (38.69%) | 82 (37.96%) |  |
| May^1^ | - | - | 64 (29.63%) |  |
| June | - | - | 31 (14.35%) |  |
| Nulliparous vs multiparous | 6 (35.29%) | 112 (56.28%) | 118 (54.63%) | 0.095 |
| Person of color vs White | 8 (47.06%) | 61 (30.65%) | 69 (31.94%) | 0.164 |
| Marital status: Married/common law vs single/other | 15 (88.24%) | 189 (94.97%) | 204 (94.44%) | 0.244 |
| College Education or higher | 15 (88.24%) | 173 (86.93%) | 188 (87.04%) | 0.878 |
| Born in Canada | 8 (47.06%) | 151 (75.88%) | 159 (73.61%) | 0.010 |
| Speaks any of the following languages (all yes/no) |  |  |  |  |
| Speaks English | 17 (100.00%) | 195 (97.99%) | 212 (98.15%) | 0.203 |
| Speaks French | 5 (29.41%) | 69 (34.67%) | 74 (34.26%) |  |
| Speaks another language | - | - | 29 (13.43%) |  |
| Combined household income above Ottawa median (120,000+) |  |  |  |  |
| Above Ottawa Median | 8 (47.06) | 104 (52.26) | 112 (51.85) | 0.596 |
| Below Ottawa Median | 8 (47.06) | 72 (36.18) | 80 (37.04) |  |
| Missing^1^ | - | - | 24 (11.11) |  |
| Dwelling is owned | 6 (35.29) | 145 (72.86) | 151 (69.91) | 0.001 |
| Children’s schedule |  |  |  |  |
| Children stopped going to school or daycare | 10 (58.82%) | 74 (37.19%) | 84 (38.89%) | 0.206 |
| Children’s schedule did not change | - | - | 14 (6.48%) |  |
| No children | 6 (35.29%) | 112 (56.28%) | 118 (54.63%) |  |
| Isolation due to COVID-19 restrictions (all yes/no) |  |  |  |  |
| No baby shower | 11 (64.71%) | 88 (44.22%) | 99 (45.83%) | 0.104 |
| Family member couldn’t come to stay with me as planned | 10 (58.82%) | 98 (49.25%) | 108 (50.00%) | 0.448 |
| Friends and family could not visit my new baby | 15 (88.24%) | 155 (77.89%) | 170 (78.70%) | 0.317 |
| Missed out on community resources | 14 (82.35%) | 169 (84.92%) | 183 (84.72%) | 0.777 |
| Safety concerns about taking my baby outside the home | 13 (76.47%) | 172 (86.43%) | 185 (85.65%) | 0.261 |
| Positive coping mechanisms (yes/no) |  |  |  |  |
| Connecting with friends/family | 14 (82.35) | 186 (93.47) | 200 (92.59) | 0.14 |
| Exercising | 12 (70.59) | 114 (57.29) | 126 (58.33) | 0.541 |
| Getting professional help^1^ | - | - | 25 (11.57) | 0.494 |
| Negative coping mechanisms (yes/no) | 15 (88.24%) | 124 (62.31%) | 139 (64.35%) | 0.032 |
| Sleeping more or less than normal | 9 (52.94%) | 93 (46.73%) | 102 (47.22%) | 0.081 |
| Over or under eating, or eating more unhealthy foods | 10 (58.82%) | 84 (42.21%) | 94 (43.52%) | 0.2 |
| Acting violently or self harm^1^ | - | - | 5 (2.31%) | 0.607 |
| Had any stress/anxiety about receiving |  |  |  |  |
| Prenatal care (yes/no) | 13 (76.47%) | 123 (61.81%) | 136 (62.96%) | 0.23 |
| Labour and delivery (yes/no) | 8 (47.06%) | 77 (38.69%) | 85 (39.35%) | 0.498 |
| Post-partum care (yes/no) | 9 (52.94%) | 96 (48.24%) | 105 (48.61%) | 0.71 |
| Feeding method in the past week |  |  |  |  |
| Breastmilk only | 8 (47.06%) | 128 (64.32%) | 136 (62.96%) | 0.314 |
| Formula | - | - | 36 (16.67%) |  |
| Combined | 5 (29.41%) | 35 (17.59%) | 40 (18.52%) |  |

1. Small cells ≤5 are suppressed

NB. The study took place between March 16th to June 16^th^, 2020.

**Appendix Table 3. Sensitivity analysis: Comparing those at risk of suicidality to those without risk of suicide and also without PPD (n=169)**

| **Variables** | **Risk of suicide** | | **Total** | **p-value** |
| --- | --- | --- | --- | --- |
|  | Yes  n=17 | No^1^  N=152 | n=169 |  |
| Maternal Age in years (median, IQR) | 33 (31-36) | 33 (30-36) | 33 (30-36) | 0.430 |
| Infant age in days at time of interview (med, IQR) | 80 (71-91) | 75 (65-90) | 75 (66-90) | 0.240 |
| Month of Delivery in 2020 |  |  |  |  |
| March | 5 (29.41%) | 29 (19.08%) | 34 (20.12%) | 0.369 |
| April | 5 (29.41%) | 55 (36.18%) | 60 (35.50%) |  |
| May | 3 (17.65%) | 48 (31.58%) | 51 (30.18%) |  |
| June | 4 (23.53%) | 20 (13.16%) | 24 (14.20%) |  |
| Nulliparous vs multiparous | 6 (35.29%) | 84 (55.26%) | 90 (53.25%) | 0.118 |
| Person of color | 9 (52.94%) | 105 (69.08%) | 114 (67.46%) | 0.178 |
| Marital status: Married/common law vs single/other | 15 (88.24%) | 143 (94.08%) | 158 (93.49%) | 0.354 |
| College Education or higher | 15 (88.24%) | 134 (88.16%) | 149 (88.17%) | 0.993 |
| Born in Canada | 8 (47.06%) | 114 (75.00%) | 122 (72.19%) | 0.015 |
| Speaks any of the following languages (all yes/no) |  |  |  |  |
| Speaks English | 17 (100.00%) | 150 (98.68%) | 167 (98.82%) | 0.634 |
| Speaks French | 5 (29.41%) | 54 (35.53%) | 59 (34.91%) | 0.616 |
| Speaks another language | 4 (23.53%) | 20 (13.16%) | 24 (14.20%) | 0.245 |
| Combined household income above Ottawa median (120,000+) | 8 (47.06%) | 77 (50.66%) | 85 (50.30%) | 0.556 |
| Children’s schedule |  |  |  |  |
| Children stopped going to school or daycare | 10 (58.82%) | 59 (38.82%) | 69 (40.83%) | 0.267 |
| Children’s schedule did not change | 1 (5.88%) | 9 (5.92%) | 10 (5.92%) |  |
| No children | 6 (35.29%) | 84 (55.26%) | 90 (53.25%) |  |
| Isolation due to COVID-19 restrictions (all yes/no) |  |  |  |  |
| No baby shower | 11 (64.71%) | 64 (42.11%) | 75 (44.38%) | 0.075 |
| Family member couldn’t come to stay with me as planned | 10 (58.82%) | 70 (46.05%) | 80 (47.34%) | 0.317 |
| Friends and family could not visit my new baby | 15 (88.24%) | 115 (75.66%) | 130 (76.92%) | 0.243 |
| Missed out on community resources | 14 (82.35%) | 125 (82.24%) | 139 (82.25%) | 0.991 |
| Safety concerns about taking my baby outside the home | 13 (76.47%) | 126 (82.89%) | 139 (82.25%) | 0.511 |
| Positive coping strategies (yes/no) |  |  |  |  |
| Negative coping patterns (yes/no) | 15 (88.24%) | 85 (55.92%) | 100 (59.17%) | 0.01 |
| Sleeping more or less than normal | 9 (52.94%) | 62 (40.79%) | 71 (42.01%) | 0.111 |
| Over or under eating, or eating more unhealthy foods | 10 (58.82%) | 55 (36.18%) | 65 (38.46%) | 0.115 |
| Had any stress/anxiety about receiving |  |  |  |  |
| Prenatal care (yes/no) | 13 (76.47%) | 85 (55.92%) | 98 (57.99%) | 0.104 |
| Labour and delivery (yes/no) | 8 (47.06%) | 52 (34.21%) | 60 (35.50%) | 0.294 |
| Post-partum care (yes/no) | 9 (52.94%) | 67 (44.08%) | 76 (44.97%) | 0.486 |
| Feeding method in the past week |  |  |  |  |
| Breastmilk only | 8 (47.06%) | 103 (67.76%) | 111 (65.68%) | 0.226 |
| Formula | 3 (17.65%) | 22 (14.47%) | 25 (14.79%) |  |
| Combined | 5 (29.41%) | 25 (16.45%) | 30 (17.75%) |  |

NB. The study took place between March 16th to June 16^th^, 2020.

1. In the main analysis there are 64 people with PPD (17 at risk of suicide and 47 with PPD alone). In this analysis, the 47 cases of PPD alone were removed to compare those with risk of suicidality (n=17) those without any form of PPD (n=169)

**Appendix Table 4. Sensitivity analyses of bivariable and multivariable log-binomial regression models to assess factors associated with risk of suicide (n=169)**

| **Co-Variables** | **RR (95% CI)** | **p-value** | **aRR (95% CI)** | **p-value** |
| --- | --- | --- | --- | --- |
| Maternal age at delivery (yrs) | 1.05 (0.95 - 1.15) | 0.342 | 1.05 (0.95 - 1.16) | 0.326 |
| Age of infant at survey completion (days) | 1.01 (0.99 - 1.04) | 0.206 | 1.01 (0.99 - 1.03) | 0.398 |
| Parity | 0.48 (0.19 - 1.24) | 0.123 |  |  |
| Person of color | 0.54 (0.22 - 1.33) | 0.182 |  |  |
| Household income under | 1.35 (0.54 - 3.40) | 0.525 |  |  |
| Negative Coping Strategies | 5.18 (1.22 - 21.91) | 0.026 |  |  |
| Pre-existing anxiety and/or depression | 3.81 (1.59 - 9.09) | 0.003 | 3.68 (1.55 - 8.76) | 0.003 |
| Feeding Method in the past week |  |  |  |  |
| Breast milk only | 0.60 (0.17 - 2.10) | 0.426 |  |  |
| Combined | 1.39 (0.37 - 5.25) | 0.628 |  |  |
| Formula |  |  |  |  |

NB. The study took place between March 16th to June 16^th^, 2020.
